# Boosting the Antibacterial Activity of Azithromycin on Multidrug-Resistant *Escherichia coli* by Efflux Pump Inhibition Coupled with Outer Membrane Permeabilization Induced by Phenylalanine-Arginine β-Naphthylamide

**DOI:** 10.3390/ijms24108662

**Published:** 2023-05-12

**Authors:** Farah Al-Marzooq, Akela Ghazawi, Lana Daoud, Saeed Tariq

**Affiliations:** 1 Department of Medical Microbiology and Immunology, College of Medicine and Health Sciences, United Arab Emirates University, Al Ain P.O. Box 15551, United Arab Emirates; 2 Department of Anatomy, College of Medicine and Health Sciences, United Arab Emirates University, Al Ain P.O. Box 15551, United Arab Emirates

**Keywords:** *Escherichia coli*, phenylalanine-arginine β-naphthylamide, azithromycin, synergy, efflux pump inhibitor

## Abstract

The global spread of multidrug-resistant (MDR) bacteria increases the demand for the discovery of new antibiotics and adjuvants. Phenylalanine-arginine β-naphthylamide (PAβN) is an inhibitor of efflux pumps in Gram-negative bacteria, such as the AcrAB-TolC complex in *Escherichia coli*. We aimed to explore the synergistic effect and mechanism of action of PAβN combined with azithromycin (AZT) on a group of MDR *E. coli* strains. Antibiotic susceptibility was tested for 56 strains, which were screened for macrolide resistance genes. Then, 29 strains were tested for synergy using the checkerboard assay. PAβN significantly enhanced AZT activity in a dose-dependent manner in strains expressing the *mphA* gene and encoding macrolide phosphotransferase, but not in strains carrying the *ermB* gene and encoding macrolide methylase. Early bacterial killing (6 h) was observed in a colistin-resistant strain with the *mcr-1* gene, leading to lipid remodeling, which caused outer membrane (OM) permeability defects. Clear OM damage was revealed by transmission electron microscopy in bacteria exposed to high doses of PAβN. Increased OM permeability was also proven by fluorometric assays, confirming the action of PAβN on OM. PAβN maintained its activity as an efflux pump inhibitor at low doses without permeabilizing OM. A non-significant increase in *acrA*, *acrB*, and *tolC* expression in response to prolonged exposure to PAβN was noted in cells treated with PAβN alone or with AZT, as a reflection of bacterial attempts to counteract pump inhibition. Thus, PAβN was found to be effective in potentiating the antibacterial activity of AZT on *E. coli* through dose-dependent action. This warrants further investigations of its effect combined with other antibiotics on multiple Gram-negative bacterial species. Synergetic combinations will help in the battle against MDR pathogens, adding new tools to the arsenal of existing medications.

## 1. Introduction

Antimicrobial resistance (AMR) is the most serious global threat to human health, projected to cause 10 million deaths per year by 2050 [1]. It is pertinent to mention that AMR is aggravated by the slow discovery and development of novel antimicrobial agents; therefore, very few new drugs are expected to be introduced for the treatment of infectious diseases [2]. One way to combat AMR is by exploiting the synergy and rejuvenation of old drugs by combining multiple drugs for treating infections caused by AMR bacteria [3]. This is beneficial as bacteria have evolved to resist existing drugs in different ways, including the expression of active efflux pumps. Initially, these efflux systems were considered as a general protective mechanism against the adverse effects of the toxic substances present in the environment. Later, bacteria evolved to use this machinery to remove antibiotics outside the cells. The extrusion of antibiotics from bacterial cells significantly lowers their clinical utility due to low intracellular drug concentration, which is an essential requirement for efficient bacterial killing [4]. Antibiotics are expelled outside the cells by membrane transporter proteins, known as efflux pumps [5]. In Gram-negative bacteria, the majority of efflux pumps contributing to AMR are tripartite pumps traversing both inner and outer bacterial membranes [6].

*Escherichia coli* is a well-known Gram-negative bacterial human pathogen that can cause a broad array of diseases, such as bacteremia, meningitis, urinary tract infections, and gastrointestinal infections [7]. A recent study reporting the global deaths attributable to and associated with bacterial AMR has identified *E. coli* as the top pathogen, with the highest mortalities due to AMR infections worldwide [8]. AcrAB-TolC is the main constitutively expressed efflux pump system in *E. coli* [7]. The tripartite AcrAB-TolC efflux pump complex belongs to one of the seven resistance–nodulation–division (RND) family efflux pumps in *E. coli* [9]. The AcrAB-TolC efflux system is notorious for its ability to extrude a broad range of antimicrobial agents, including macrolides and other drugs such as penicillins, cephalosporins, tetracyclines, lincosamides, etc. [9]. Upregulation of acrAB operon in *E. coli* has been associated with high levels of resistance to multiple antibiotics, such as tetracycline and ciprofloxacin [10]. A recent study demonstrated that the inactivation of AcrA or AcrB in *E. coli* led to decreased bacterial viability in macrophages [11], which indeed reflects the importance of efflux pump inhibitors to exploit AcrAB function in bacterial pathogens, as the AcrAB complex has been proven to have a role in virulence, pathogenesis, and survival within infected host cells, in addition to its role in aggravating AMR.

Azithromycin (AZT) is a macrolide that targets bacterial protein synthesis [12]. Besides its antibacterial activity, AZT has anti-inflammatory and immunoregulatory properties that complement its activity in managing infections [13]. Studies have shown that prolonged AZT therapy at low doses can improve the clinical outcome in patients with respiratory tract infections such as cystic fibrosis [14]. Unfortunately, AZT has low activity against *Enterobacterales*, including *E. coli*, owing to its poor membrane penetration [15]. In our previous work, we proved that the activity of AZT can be improved by synergy with an outer membrane (OM) permeabilizing agent, namely, polymyxin B nonapeptide (PMBN). It increased the influx of AZT, hence improving the antibacterial activity of the drug [16]. Therefore, we decided to investigate whether decreasing the efflux of the drug would also improve the antibacterial activity of AZT. Phenylalanine-arginine β-naphthylamide (PAβN) was selected for use in this study. PAβN is a peptidomimetic compound which was originally described as a broad-spectrum efflux pump inhibitor [5]. Recent reports have indicated that PAβN can also perturb bacterial OM, causing an increase in permeability [17,18]. This motivated us to investigate the effect of this molecule on our clinical strains, especially because mechanistic studies for PAβN action on bacteria are lacking. Therefore, this study was conducted with the aim to test the effect of PAβN on the antibacterial activity of AZT against a group of *E. coli* strains. The study also aimed to investigate the killing kinetics of the synergistic combinations and the mechanism of improved antibacterial activity, phenotypically and genotypically, by visualizing the ultrastructural changes in the treated bacteria by transmission electron microscopy.

## 2. Results

**Figure 1:**
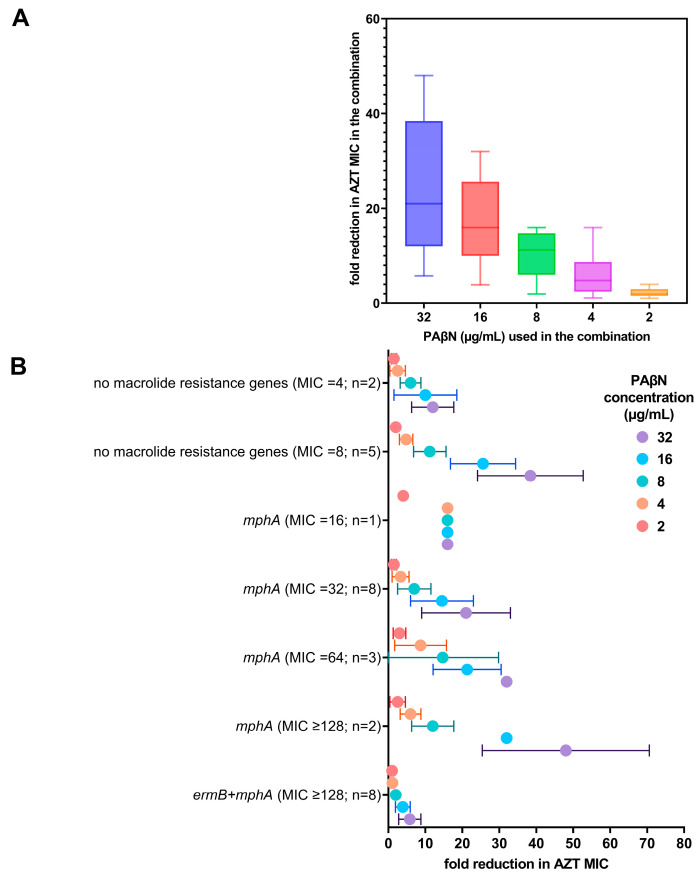
Reduction in AZT MIC when used in combination with PAβN. (**A**) Fold reduction of AZT MIC in combination with different doses of PAβN (2–32 µg/mL). The fold reduction shown is the mean ± SD of all the strains regardless of the AZT MIC, while (**B**) shows the relation of AZT MIC (alone) with the genotypes of the strains with fold reduction in AZT MIC when used in combination with different doses of PAβN. n represents the number of strains per group.

Initially, a total of 56 *E. coli* strains were tested for their susceptibility to different antibiotics and screened for the most important macrolide resistance genes and efflux pumps. Antibiotic susceptibility data and detected genes in all the strains are provided in Supplementary Table S1. In summary, the collection included only 4 (7.1%) susceptible bacteria, while the remaining 52 bacteria (92.9%) were multidrug-resistant (MDR), including some extensively drug-resistant (XDR) bacteria (n = 16; 28.6%) with various resistance profiles. As for AZT, a total of 34 strains (60.7%) were non-susceptible, with MIC ranging from 32 to ≥128 µg/mL. Of these, 8 strains carried both the *ermB* and *mphA* genes, while 26 strains carried the *mphA* gene alone. No other macrolide resistance genes and macrolide efflux pumps were detected. The next step was selecting 29 strains for synergy experiments based on their genotypes and susceptibility to AZT. The collection included 10 AZT-resistant strains with very high MIC (≥128 µg/mL), carrying either the *mphA* gene alone (n = 2) or with the *ermB* gene (n = 8). Another 12 AZT-resistant strains with lower MICs (16–64 µg/mL) were positive for the *mphA* gene alone, while the susceptible strains (n = 7) were devoid of macrolide resistance genes (MIC = 4–8 µg/mL). The selected bacteria, their genotypes, and the results of AZT/PAβN synergy are summarized in Figure 1.

PAβN did not have any antibacterial activity when used alone (MIC > 128 µg/mL). When PAβN was used in combination with AZT, there was a dose-dependent reduction in the MIC of AZT in the combination, as shown in Figure 1A. FICI ranged between 0.094–0.1875, being less than 0.5, which confirmed the synergistic activity of PAβN with AZT. The FICIs of the tested bacteria are shown in the supplementary data file (Table S2).

When the genotypes of the strains were correlated with AZT MICs, strains expressing both *ermB* and *mphA* were found to have higher MICs (MIC ≥ 128 µg/mL) and exhibited a poor response to the combination, while strains harboring *mphA* alone demonstrated a significant reduction in the MIC (*p* < 0.05), even in strains with high initial AZT MICs (≥128 µg/mL), as shown in Figure 1B. When high doses of PAβN (16–32 µg/mL) were used, the reduction in AZT MIC was most significant (4–64-fold), depending on the initial MIC of AZT without any synergistic additive, as shown in Figure 1B.

**Figure 2:**
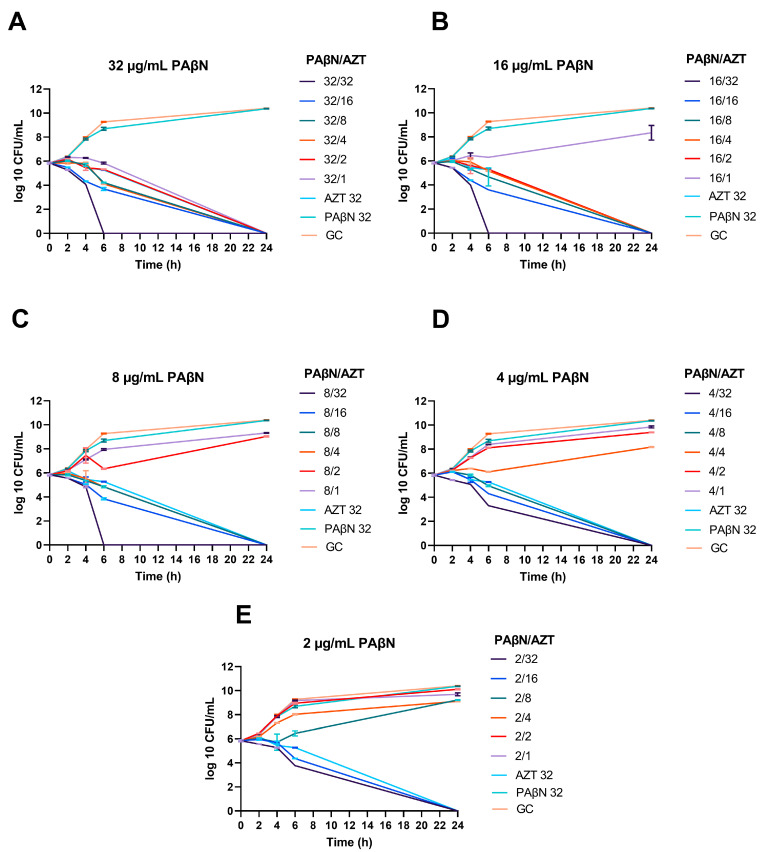
Synergistic effect of PAβN with AZT, demonstrated by time-kill graphs. A selected *E. coli* strain (CDC AR-0346) is shown. Different synergistic combinations of PAβN (2–32 µg/mL) and AZT (1–32 µg/mL) were tested (**A**–**E**). PAβN and AZT (32 µg/mL) were used as controls in addition to an untreated growth control (GC). PAβN had no antibacterial activity when used alone, while synergistic combinations with AZT ≤ ½ X MIC killed the bacteria, depending on the concentration of PAβN used. The data shown represent the mean value of duplicates from two independent experiments ± SD.

Time-kill assays were performed for selected *E. coli* strains (n = 4), to determine the time needed for bacterial killing by the synergetic combinations. The time-kill graphs are included in the supplementary file (Figures S1–S3), while Figure 2 shows a representative XDR strain that is resistant to colistin (CDC-AR-0346).

As seen in the time-kill graphs, the highest concentration of PAβN (32 µg/mL) alone did not have any effect on bacterial growth compared to the growth control (untreated culture), while it caused bacterial killing when used in combination with AZT at different doses. When PAβN was used at the highest concentration tested (32 µg/mL), bacterial death could be achieved even at concentrations lower than AZT MIC for this strain (32 µg/mL).

Noteworthy, killing could be achieved in a shorter time (6 h) when PAβN was used at 8–32 µg/mL with 32 µg/mL of AZT. On the other hand, while using AZT alone at the same dose (32 µg/mL) could kill the bacteria over a prolonged time, reaching up to 24 h. Killing at 6 h was seen only in one strain (CDC-AR-0346), which was the only strain in our collection with resistance to colistin due to the presence of the *mcr-1* gene. On the other hand, when lower doses of PAβN were used, the effect of the synergistic combination was less, and caused killing after 24 h of treatment, as shown in Figure 2.

The same results were obtained in the other strains tested (n = 3), but killing was achieved after 24 h of treatment with the synergistic combinations, as shown in time-kill graphs (Figures S1–S3). In summary, bacterial death was achieved at lower doses of AZT (≥1/16 X MIC) when a high dose of PAβN (8–32 µg/mL) was used in the combinations. Lower doses of PAβN caused bacterial death at ≥1/8 X and ≥1/4 X MICs of AZT when used with 4 and 2 µg/mL of PAβN, respectively.

**Figure 3:**
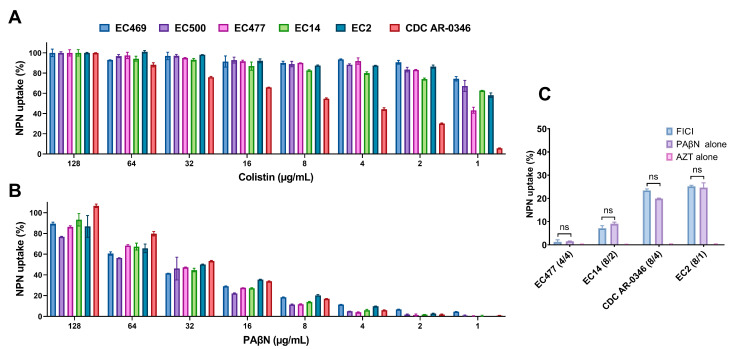
Assessment of outer membrane permeability by 1-N-phenylnaphthylamine (NPN) assay in six selected *E. coli* strains. Data shown represent % of NPN uptake by bacteria treated with a serial dilution (0.5–128 μg/mL) of colistin, used as a positive control (**A**), and PAβN (**B**). Four selected bacteria treated with the synergetic combination of PAβN and AZT (concentrations reported between the brackets, respectively) at FICI in comparison with treatment with a single agent are shown in (**C**). ns represents a non-significant difference. The EC469 and EC500 strains were not included in figure (**C**) as synergy was not effective on these two strains, which carried both *ermB* and *mphA*, in contrast to the other strains, which carried the *mphA* gene only.

To assess the OM integrity of bacteria treated with PAβN, we measured the uptake of the fluorescent probe 1-N-phenylnaphthylamine (NPN) in six selected strains treated with a serial dilution of PAβN (1–128 µg/mL). As shown in Figure 3, the uptake of NPN was dose-dependent when the bacteria were treated with colistin as a positive control (A) or PAβN (B). Significantly higher uptake of NPN (>50% uptake) was achieved when high concentrations of PAβN (64–128 µg/mL) were used. Noteworthy, the strain CDC-AR-0346 (resistant to colistin) exhibited the highest uptake of NPN at PAβN concentrations ≥ 32 µg/mL compared to other strains. This contrasted with the findings seen when the same strain was treated with colistin, as the NPN uptake was the lowest (Figure 3A) compared to the colistin-susceptible strains.

Figure 3C demonstrates four selected strains treated with PAβN and AZT synergetic combinations at FICI in comparison with treatment with a single agent. By looking at the % NPN uptake in the strains with different treatments, the AZT/PAβN combination was almost equivalent to PAβN alone, as the difference in % NPN uptake was not statistically significant (*p* > 0.05). AZT did not have any effect on NPN uptake.

**Figure 4:**
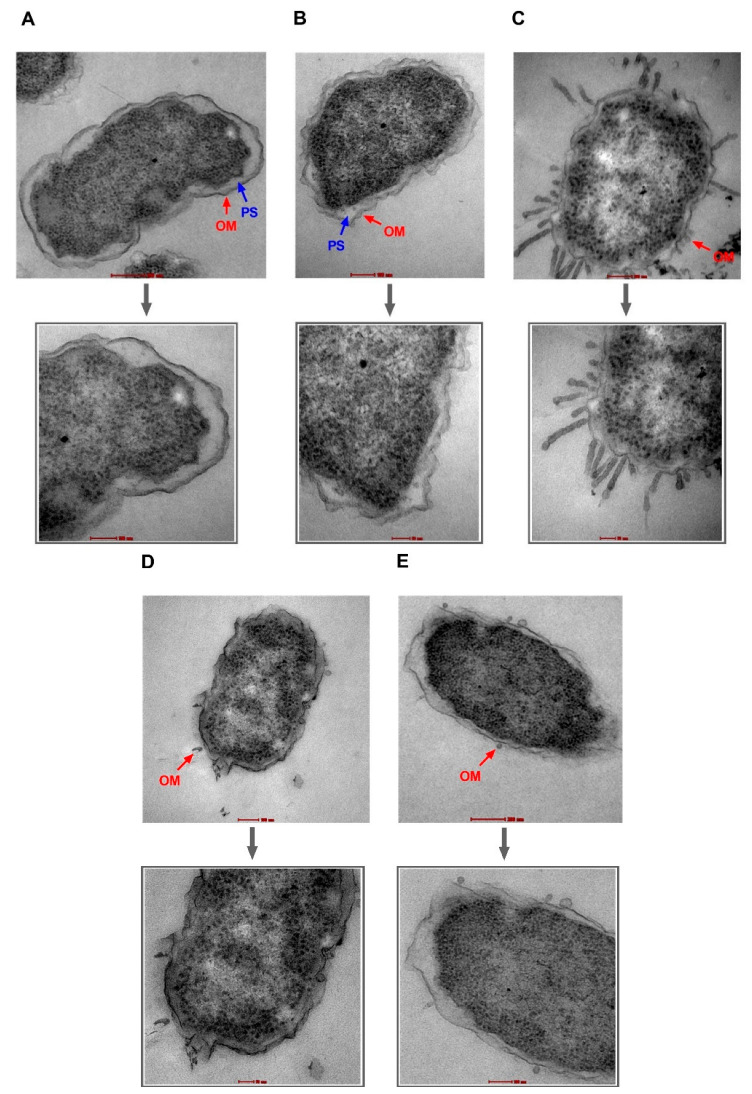
Transmission electron micrographs of *E. coli* strain EC477. Untreated cells (**A**) and cells treated with AZT—128 µg/mL (**B**) are shown with intact outer membranes (OMs) and clear periplasmic spaces (PS). Damage to the OM was obvious in bacterial cells treated with the positive control (colistin—2 µg/mL) (**C**), PAβN alone—64 µg/mL (**D**), and PAβN—64 µg/mL plus AZT—128 µg/mL (**E**). Magnified sections are shown below each figure to demonstrate an intact OM (**A**,**B**) versus a damaged OM (**C**,**E**) caused by the treatment. Magnification scale is 50–200 nm.

**Figure 5:**
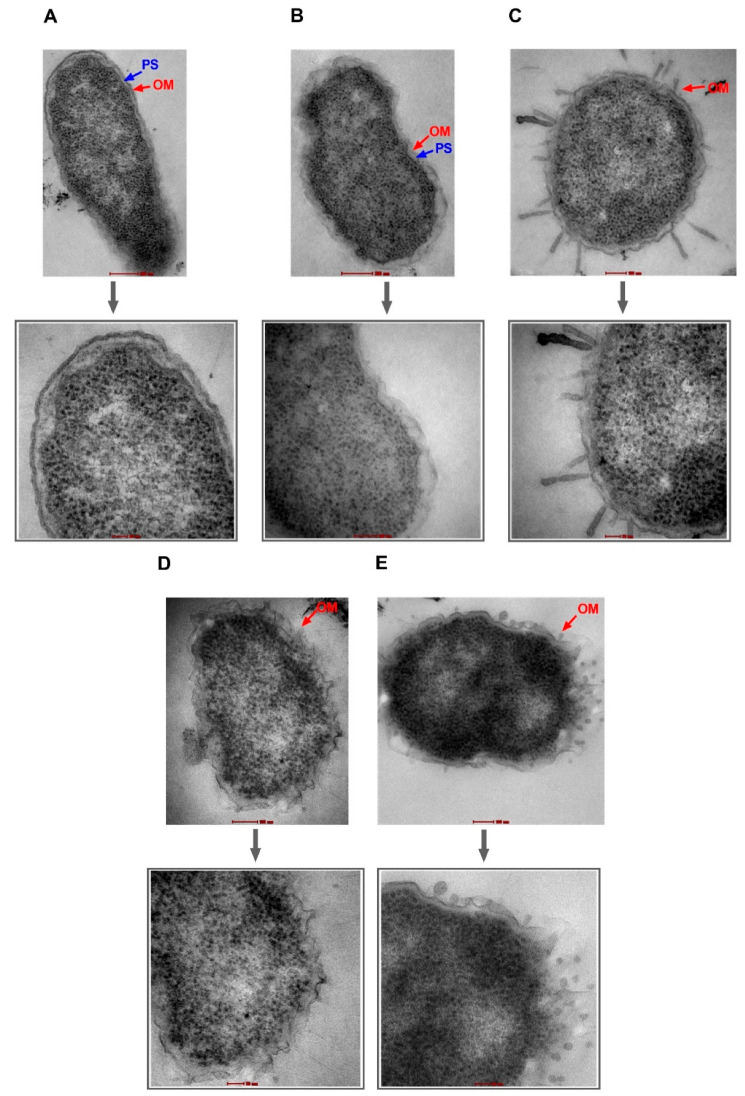
Transmission electron micrographs of *E. coli* strain EC500. Untreated cells (**A**) and cells treated with AZT—128 µg/mL (**B**) are shown with intact outer membranes (OMs) and clear periplasmic spaces (PS). Damage to the OM was obvious in bacterial cells treated with the positive control (colistin—2 µg/mL) (**C**), PAβN alone—64 µg/mL (**D**), and PAβN—64 µg/mL plus AZT—128 µg/mL (**E**). Magnified sections are shown below each figure to demonstrate an intact OM (**A**,**B**) versus a damaged OM (**C**,**E**) caused by the treatment. Magnification scale is 50–200 nm.

NPN uptake in bacteria treated with high doses of PAβN suggests damage to OM facilitating NPN entry. If this is true, then the same strategy can be used by AZT to enter the bacterial cell. To verify these findings, TEM was used to visualize two selected bacterial strains, specifically EC477 with *mphA* alone and a good response to the synergistic AZT/PAβN combination in addition to strain EC500, with both the *ermB* and *mphA* genes and a poor response to the AZT/PAβN combination. Figure 4 and Figure 5, show strains EC477 and EC500, respectively.

As shown in Figure 4 and Figure 5, bacterial strains treated with PAβN alone or in combination with AZT exhibited compromised outer membranes. Protrusions, blebs, or short threads were seen either detached or attached to the OM in these treated cells. However, the magnitude of damage was less compared to the positive control (colistin), which is well known as a membrane-damaging agent causing longer threads and more clear deformation and defects in the OM despite treatment with a low dose (2 µg/mL).

Notably, cells treated with AZT exhibited some irregularities in the OM, which seemed to remain intact. Damage caused by PAβN alone or in combination with AZT was similar in the two strains (EC477 and EC500), although the latter strain was a poor respondent to the AZT/PAβN combination (shown in Figure 1, with strains having MIC ≥128 µg/mL and both the *ermB* and *mphA* genes).

**Figure 6:**
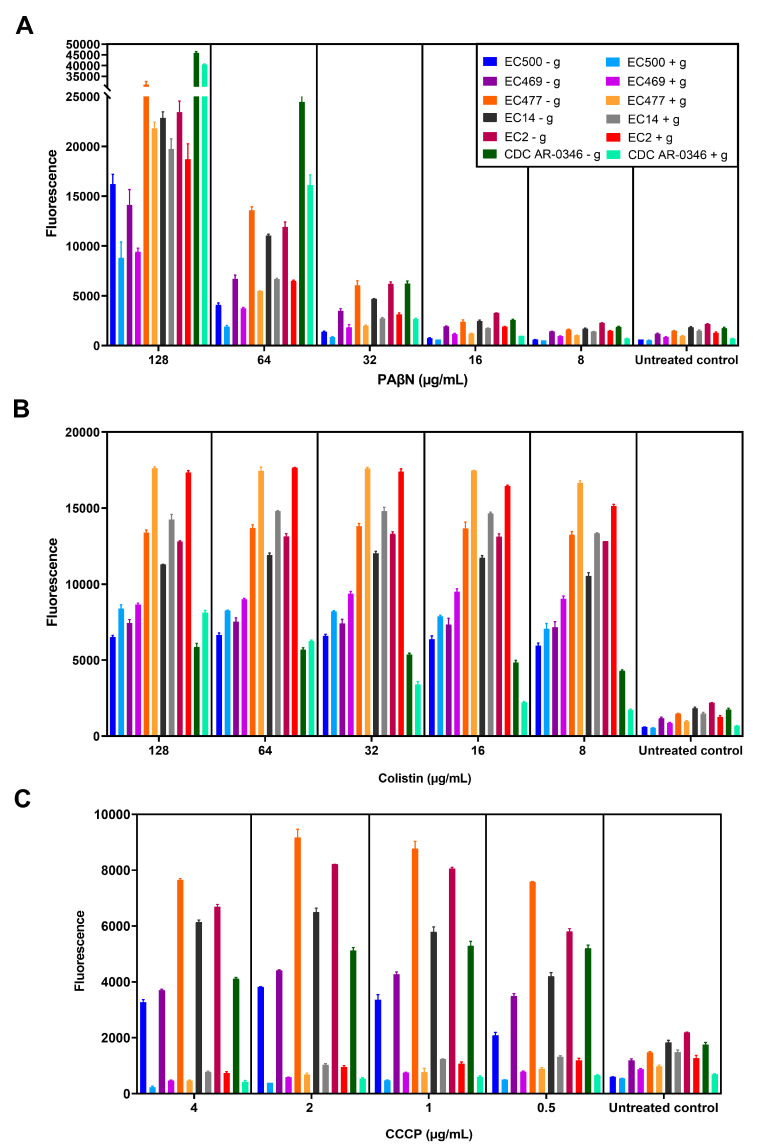
NPN efflux pump assay. Six selected bacterial strains (shown in the figure legend) were treated with serial dilutions of PAβN (**A**), colistin (**B**), or CCCP (**C**), either without adding glucose (marked with - g) or with glucose (marked with + g). The fluorescence level shown is after 5 min of incubation of bacteria pretreated with the test agents, after adding glucose to the appropriate cultures while keeping glucose-untreated cultures as comparators.

After proving that OM damage was induced by PAβN with and without AZT, PaβN’s effect on efflux pumps was examined in *E. coli* using a special NPN assay, both with and without the addition of glucose. Figure 6 shows the NPN fluorescence of six selected strains treated with different doses of PAβN (A), colistin (B), or CCCP (C). Data shown in this figure represent the NPN fluorescence recorded after 5 min of adding glucose to the indicated cultures, keeping glucose-untreated cultures as comparators.

As shown in Figure 6A, PAβN caused a significant increase in NPN fluorescence when high doses (64–128 µg/mL) were used compared to lower doses (≤32 µg/mL), whether the culture was treated with glucose or not. NPN fluorescence (dye uptake) was higher in bacterial strains responding to the synergistic combinations, and was lower in non-responding strains (EC500 and EC469), but the difference was non-significant (*p* > 0.05). Notably, there was a decrease in the fluorescence (1.7 ± 0.5 folds) after adding glucose, but it was not statistically significant compared to cells not treated with glucose. The same was noted in the untreated control (1.6 ± 0.5-fold reduction).

In contrast (as shown in Figure 6B), bacteria treated with colistin (OM-damaging agent) showed a significant increase (*p* < 0.05) in NPN fluorescence after treatment with glucose (1.2 ± 0.2 folds increase), with the exception of strain CDC-AR-0346, which was resistant to colistin, contrary to the other strains. However, NPN uptake was reduced in strain CDC-AR-0346 after adding glucose to cells treated with ≤32 µg/mL of colistin, as seen in PaβN-treated cells. As for cells treated with CCCP, which was used as a standard efflux pump inhibitor (Figure 6C), there was a significant reduction in the fluorescence after treatment with glucose (8.3 ± 3.3 folds reduction) compared to glucose-untreated cells. It is noteworthy that the reduction in fluorescence in PaβN-treated cells after adding glucose (1.7 ± 0.5 folds) was less compared to CCCP.

**Figure 7:**
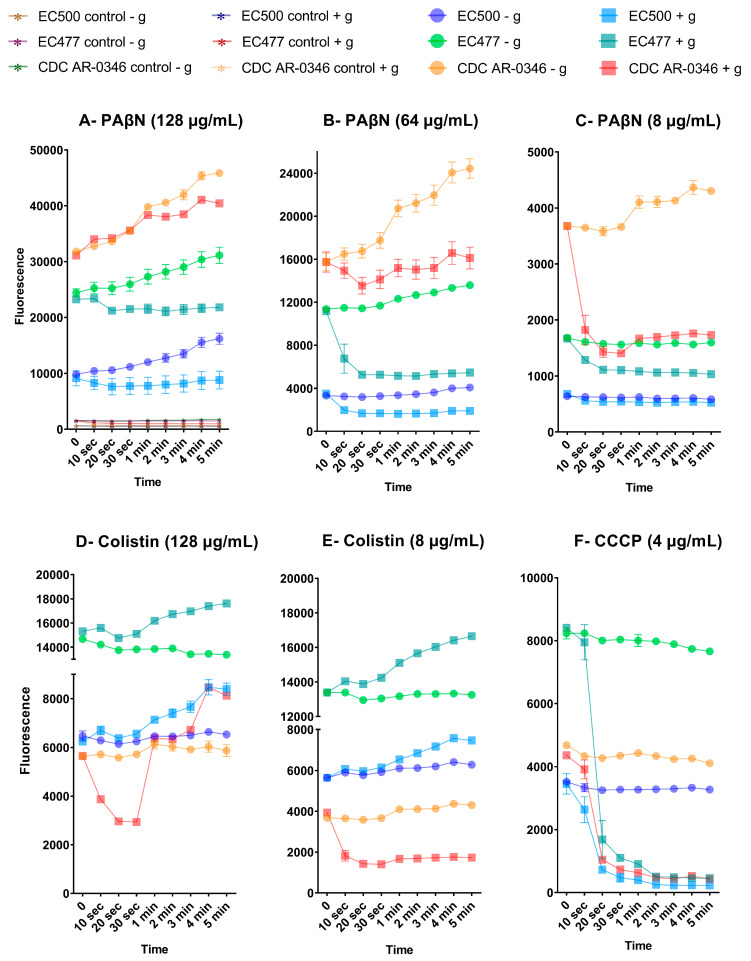
Change in NPN fluorescence over time, with and without glucose treatment, after exposure to PAβN (**A**–**C**), colistin (**D**,**E**), and CCCP (**F**). Fluorescence was recorded over 5 min of incubation time. Values are expressed as mean ± standard deviation. Untreated controls are shown in figure (**A**), and were not included in the other figures to avoid overlapping with other curves with low fluorescence levels. - g: without glucose; + g: with glucose.

Efflux pump activity was examined and followed up in real-time by monitoring the change in NPN fluorescence over 5 min of treatment, as shown in Figure 7, which depicts three selected strains based on their responses to PAβN, namely strains CDC-AR-0346, EC477, and EC500. Bacteria were treated with high doses (64–128 µg/mL) and low doses (8 µg/mL) of PAβN (Figure 7A–C) to show the difference in their effect. Additionally, both a high dose (128 µg/mL) and a low dose (8 µg/mL) of colistin (Figure 7D,E) were used. A single dose of CCCP (4 µg/mL) was shown (Figure 7F), while other doses were not, as a non-significant difference was detected among them (as seen in Figure 6C). As shown in the figures, fluorescence was compared in bacteria incubated with and without glucose treatment.

An interesting observation was the difference in fluorescence among the bacteria treated with PAβN, which were ranked based on their response to AZT/PAβN synergy, categorized as CDC-AR-0346 (early killing and good response to synergy), EC477 (good response to synergy), and EC500 (poor response to synergy). This was not seen with CCCP or colistin-treated cultures. Colistin-resistant strain (CDC-AR-0346) showed the least fluorescence after exposure to colistin, and was unique in its response to all types of treatment. When this strain was treated with a high dose of PAβN (128 µg/mL), it exhibited a progressive increase in fluorescence over time in both glucose-treated and -untreated cells; however, more fluorescence was detected in the latter. This contrasts with the other strains, as cells treated with glucose maintained a steady level of fluorescence, while glucose-untreated cells showed a progressive increase in fluorescence over time when treated with 128 µg/mL of PAβN. At lower doses of PAβN, there was a clear decline in the fluorescence level quickly (10 s) after adding glucose compared to untreated cells, which showed a slight increase or steady fluorescence level when treated with 64 and 8 µg/mL, respectively.

For CDC-AR-0346, the decline in the fluorescence level after glucose treatment was seen only when the bacteria were treated with a low dose of PAβN (4 µg/mL), although cells seemed to be recovered after 1 min, with an increase in fluorescence. This was also shown when the strain was treated with a high dose of colistin (128 µg/mL), but not with a low dose (8 µg/mL); it exhibited a marked reduction in fluorescence after treatment with glucose. This contrasts with the other strains, which exhibited an increase in fluorescence after adding glucose to colistin-treated cells regardless of the dose. As for CCCP-treated cells, there was a significant reduction in fluorescence after 20 s of adding glucose, which was consistent for all the strains.

In order to explore changes at the molecular level for the three components of the efflux pump (AcrAB-TolC), we conducted gene expression studies for seven selected strains. These encompassed strains that were susceptible to AZT (n = 2) and others which were resistant to it, both those with a poor response (n = 2) as well as with a good response (n = 3) to the synergistic combinations. Based on the results of the time-kill study and considering the fact that AZT can significantly lower bacterial growth after 6 h of treatment, we decided to treat the bacterial cultures for 6 h with a fixed concentration of PAβN (128 μg/mL) and 2X MIC of AZT for each strain, plus the combination of PAβN and AZT at the same dose. Real-time PCR was used to measure the expression of *acrA*, *acrB*, and *tolC* in the treated cells, normalized to the expression level of untreated cells.

**Figure 8:**
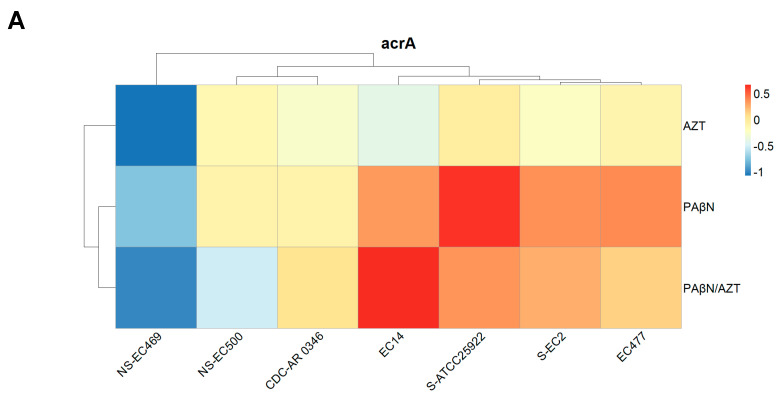

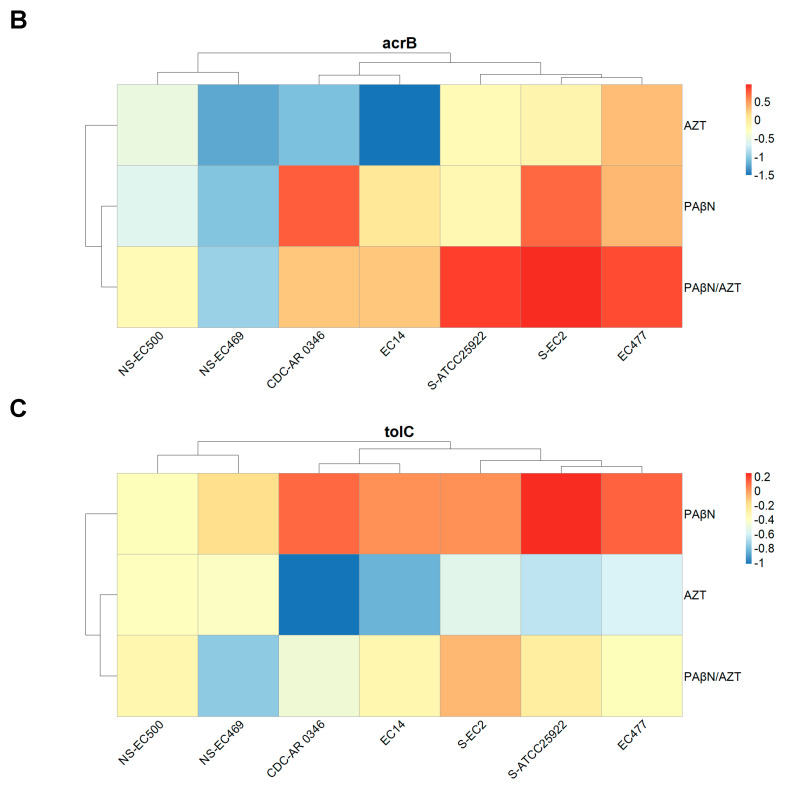
Relative expression of *acrA* (**A**), *acrB* (**B**), and *tolC* (**C**) in selected bacterial strains treated with AZT, PAβN alone, and in combination. Some strains are marked with NS (no synergy between AZT and PAβN) or S (susceptible to AZT), while non-marked strains are those which were resistant to AZT and responded well to the synergistic action of PAβN.

As shown in Figure 8, strains resistant to AZT with poor response to the synergetic combination (marked with NS) showed unique expression profiles for all three genes, which were lower than all the other strains. Gene expression was less in the bacteria treated with AZT compared to other forms of treatment, and the highest expression was noted for cultures treated with PAβN alone, especially for *acrA* and *tolC*. On the other hand, *acrB* expression was highest in cultures treated with the combination. There were strains with unique expression patterns, such as strain CDC-AR-0346. For *acrA*, the expression levels did not vary despite treatment with different agents, while expression levels were higher in PaβN-treated cells for both *acrB* and *tolC.*

The difference in gene expression was not statistically significant when the three treatments were compared for *acrA* and *acrB*, but it was significant for *tolC* (*p* < 0.05), as the group treated with PAβN exhibited the highest expression, which is also shown in Figure 8C.

When the expression levels of the three genes were compared to each other’s, the difference was not statistically significant (*p* > 0.05). Despite the observed variations, the difference was not statistically significant between strains with various levels of susceptibility to AZT.

## 3. Discussion

Efflux pumps endanger the activity of multiple antibiotics that fail to eradicate bacteria due to low concentrations at the target site. Therefore, it is believed that efflux pump inhibitors can be beneficial adjuvants to existing antibiotics. In the present study, we investigated the activity and mechanism of action of an efflux pump inhibitor (PAβN) and proved its effectiveness in improving the activity of AZT from the macrolide class of antibiotics. Combinations of AZT with PAβN were successful in killing *E. coli* strains resistant to multiple antibiotics from different classes, including highly potent drugs reserved for use as a last resort, such as colistin and carbapenems, aside from standard antibiotics commonly prescribed to treat infections caused by *E. coli* such as ciprofloxacin and trimethoprim/sulfamethoxazole, among others. Indeed, there is an urgent need for potent drug combinations to tackle this resistant pathogen, which causes high mortality rates globally [8].

Time-kill studies confirmed the bactericidal activity of the combinations, with a significant reduction in AZT MIC. This is in agreement with previous studies proving the effectiveness of PAβN as an adjuvant with other antibiotics, such as β-lactams [18], erythromycin, chloramphenicol, and nalidixic acid [19]. The activity of the AZT/PAβN combination was influenced by the genotype of the tested strains, in terms of the presence of macrolide resistance genes, which was also found in our previous study which tested the synergy between PMBN and AZT [16]. The presence of the *ermB* gene rendered AZT/PAβN combinations ineffective, as this gene encodes a methylase enzyme that modifies the ribosomal target of AZT, conferring high-level resistance to macrolides due to poor drug–target interactions [15,20]. In contrast, AZT/PAβN combinations were effective if *mphA* gene was present alone, as this gene encodes the macrolide 2′-phosphotransferase I (MphA) enzyme responsible for macrolide inactivation [20]. This is in line with our previous study [16], in which we concluded that high intracellular concentrations of AZT may overpower the inactivating enzyme produced by the bacteria and, thus, aid in restoring the antibacterial activity of AZT. This is probably related to the mechanism of action of PAβN, as this molecule was initially described as a broad-spectrum efflux pump inhibitor, but recent reports indicated its ability to breach bacterial membranes [5]. This supports our hypothesis that PAβN acts by increasing the influx and decreasing the efflux of antibiotics. Indeed, molecules such as PAβN are very attractive due to their capability to achieve both efflux inhibition and OM permeabilization, which can ultimately guarantee high intracellular concentration at the antibiotic target site.

The effect of PAβN was shown to be concentration-dependent, as low concentrations of PAβN were believed to cause efflux pump inhibition while high concentrations were capable of destabilizing bacterial membranes, adding to the inhibitory function of PAβN on efflux pumps [19]. We agree with the findings of the previous studies, as we found that PAβN improved the antibacterial activity of AZT in a dose-dependent manner, with a more potent synergistic effect achieved with higher doses. Furthermore, we have shown, by NPN uptake assay and efflux pump assay, that the effect of PAβN is more significant at high doses (64–128 µg/mL). This was further supported by the TEM results, in which damage to OM was clearly visible after treatment with PAβN both with and without AZT. To the best of our knowledge, this is the first study demonstrating the disruption of OM by examining the bacteria under TEM after treatment with PAβN. It is noteworthy that cells treated with AZT showed some irregularities in the OM, probably due to the chemical stress imposed by the high dose of AZT (128 µg/mL) used in the experiment. In contrast to the positive control (colistin), which caused massive OM damage, OM disruption caused by PAβN manifested as short threads and protrusions. Interestingly, the effect on OM was similar in strains both responding and non-responding to AZT/PAβN combinations. However, an NPN efflux pump assay proved quantitatively that strains responding poorly to AZT/PAβN combinations were less affected by PAβN, as the uptake of the fluorescent dye was less than the other strains responding well to the combination. This indicates less efflux pump inhibition and/or OM permeabilization in non-responding strains.

The inhibitory effect of PAβN on efflux pumps was examined using an NPN assay both with and without the addition of glucose, which presumably produces the proton motive force across the cytoplasmic membrane, increasing the expulsive activity of efflux pumps [7]. The use of fluorogenic dyes such as NPN has been considered a good approach to studying the activity of efflux pumps [21]. NPN is an uncharged lipophilic molecule that fluoresces strongly in nonpolar hydrophobic environments, such as the lipid bilayers of biological membranes. Furthermore, NPN is a substrate of RND efflux pumps [5]. Therefore, monitoring the efflux of NPN in live cells was carried out in this study similarly to other investigators working on *E. coli* [19]. Upon the addition of an efflux pump inhibitor, these dyes become trapped inside the cells, leading to high fluorescence [22]. This was clearly seen in the tested strains by the significant difference in fluorescence between untreated cells and PaβN-treated cells, which exhibited higher fluorescence due to higher NPN uptake. Notably, NPN uptake and fluorescence intensity were highest in strains treated with high doses (64–128 µg/mL) of PAβN. PAβN did not only inhibit efflux pumps, but also permeabilized the OM, which facilitated the entry of NPN, leading to increased intracellular levels [19].

The addition of glucose caused a non-significant reduction in fluorescence compared to another efflux pump inhibitor (CCCP) used as a control. In contrast to PAβN, adding glucose to the cultures treated with CCCP caused a significant reduction in the fluorescence, which was also shown in previous studies [7]. It was shown that the dye is able to diffuse into CCCP-treated cells, making them fluorescent, while the consequent addition of glucose leads to a concomitant reduction in cell-associated fluorescence. This was attributed to the reactivation of the efflux pumps by energization with glucose, which was able to reverse the effect of CCCP by restoring the proton motive force across the cytoplasmic membrane, leading to dye extrusion out of the cells [7]. Previous studies also reported a sharp decline in NPN fluorescence in CCCP-treated cells after glucose addition [19], which was noted in this study. Molecules such as CCCP act solely as efflux pump inhibitors, which is not the case for PAβN. Thus, the reduction in fluorescence in PaβN-treated cells after adding glucose was minor, probably due to the OM-permeabilizing effect when used in high doses. A slight elevation in NPN fluorescence likely resulted from increased NPN influx rather than its reduced efflux [22]. In this case, OM damage can ensure the continuous influx of the dye into the cells even if efflux pumps are activated after energization with glucose. Interestingly, monitoring the fluorescence over time demonstrated a progressive increase in fluorescence in cells untreated with glucose, i.e., no efflux activation, reflecting continuous NPN entry into the cells due to progressive OM damage. There was a decay of fluorescence in bacteria treated with lower doses of PAβN after energization with glucose. This is in line with a previous study reporting that PAβN acts solely as an efflux pump inhibitor at low doses [19].

Paradoxical results were seen in colistin-treated cultures after adding glucose, as a significant increase in NPN fluorescence was noted, except for in strain CDC-AR-0346 (colistin-resistant), which showed less fluorescence after adding glucose to cells treated with ≤32 µg/mL of colistin. As an OM-damaging agent, colistin probably caused a complete destruction of the bacterial membranes, leading to increased entry of NPN to the cells after adding glucose. This is in agreement with a previous study which reported that polymyxins as PMBN caused a greater damaging effect on OM than PAβN using an NPN assay [19].

In a previous study, PAβN caused a sustained increase in NPN fluorescence in glucose-treated *E. coli* cells after the addition of PaβN, which was attributed to the inhibitory effect of PAβN on efflux pumps. Nevertheless, NPN fluorescence also increased in the ΔacrAB strain (with *acrAB* gene deletion), suggesting that OM permeabilization could be the cause of this incline in fluorescence with a lack of efflux pump in this strain [19]. Another group of researchers also reported that 32 μg/mL of PAβN was sufficient to significantly elevate fluorescence in *E. coli* strains lacking AcrB or TolC [23]. Collectively, all of these reports and our study confirm the activity of PAβN on OM. Furthermore, a recent study provided proof of PaβN’s action on OM by in vitro random mutagenesis, whereby the target site of PAβN was identified to be in the lipopolysaccharide (LPS) layer of OM, indicating that PaβN’s activity on OM contributes to its antibiotic-sensitizing potency [17].

It is important to mention that some antibiotics (including macrolides and other hydrophobic antibiotics, such as aminoglycosides) can cross the OM through lipid-mediated pathways [24]. This can explain the findings of our study, as AZT (a macrolide) entry into the cell was facilitated by PAβN due to enhanced influx through disrupting the OM permeability barrier and inhibiting the active efflux pumps [25]. Previous studies have reported that mutational alteration of the LPS structure can also cause perturbation of the OM permeability barrier [25]. We agree with these reports, as we found one strain (CDC-AR-0346) to be very unique due to the presence of the *mcr-1* (mobile colistin resistance) gene, leading to colistin resistance. This strain was killed in 6 h after treatment with the AZT/PAβN synergetic combination, compared to the 24 h needed to kill other bacteria. Even when AZT was used with PMBN (OM-damaging agent) on the same strain in our previous work, delayed killing was observed after 24 h of treatment [16]. It also exhibited higher NPN uptake than the other strains treated with PAβN in contrast to its response to colistin, which was less significant, reflecting a more powerful OM-permeabilizing effect of PAβN than colistin. By looking at the genomic content of this strain, the *mcr-1* gene encodes an LPS-modifying enzyme; thus, this strain has modified lipid A with phosphoethanolamine. The addition of a phosphoethanolamine moiety to lipid A can alter the structure of lipid A in the OM, leading to a lower growth rate, cell viability, and competitive ability [26]. Multiple studies have confirmed that *mcr-1* imposes a fitness cost on *E. coli*. High expression of *mcr-1* protects the bacteria against polymyxins, but compromises its fitness and membrane structural integrity [26]. In addition, it changes the membrane’s permeability and reduces its resistance to hydrophobic antibiotics [24]. A previous study demonstrated that *mcr-1* was associated with a loss of the cell membrane integrity and a reduction in MICs of multiple antibiotics, such as gentamicin, kanamycin, and rifampicin [24]. A recent study reported that *mcr-1* overexpression increased OM permeability and caused membrane depolarization, which was supported by the findings of transcriptomic studies demonstrating the downregulation of multiple genes involved in LPS core and O-antigen biosynthesis [27].

Colistin is considered the last-resort antibiotic for treating XDR bacteria when other antibiotics are deemed ineffective. Indeed, the finding of our study is very promising, as PAβN can target the OM of *mcr-1* expressing strains, destabilize it, and increase its permeability, leading to an influx of other antibiotics that can be efficient in killing the bacteria. These findings must be confirmed by testing other strains harboring this gene, which could not be achieved in this study due to a lack of strains carrying the *mcr-1* gene. Additionally, PAβN’s effect on strains with other mechanisms causing colistin resistance associated with LPS alterations [28] should be explored.

In this study, we also investigated the expression of efflux pump genes for AcrAB-TolC, which is the archetype RND efflux pump system in *E. coli* [29]. AcrAB-TolC is a tripartite transporter that captures substrates from the periplasm and effluxes them across OM, out of the cell. AcrB is an inner membrane protein that also extends into the periplasm, responsible for drug specificity and energy transduction of the efflux pump; thus, it is considered a proton motive force-driven inner membrane transporter [30]. AcrA is a periplasmic adaptor protein located in the periplasm [31]. TolC is the OM channel for the pump responsible for the transport of substrates towards the extracellular environment [11]. In *E. coli*, the OM factor TolC is considered as a shared transporter responsible for different processes, including the expulsion of metabolites, acid tolerance, cell membrane integrity, virulence, and antibiotic resistance [29]; thus, one of the major physiological functions of this efflux system is to protect *E. coli* against cytotoxic substances. It has been reported that *acrAB* transcription is higher under stressful conditions such as exposure to 4% ethanol [32]. Adding to that, *acrAB* expression was shown to be high in MDR mutants of *E.coli*, confirming the link between AMR and efflux pump expression [32]. Because the majority of the strains tested in this study were MDR, high expression levels were expected. Despite exposure to an efflux pump inhibitor, the expression level was high if we compare it to cells treated with AZT. It is possible that AZT suppressed the gene expression in general as part of its activity as a protein synthesis inhibitor by binding to the ribosomes, as it acts by blocking the peptide exit channel of the 50S ribosomal subunit through interaction with the 23S rRNA [33]. Previous studies have also shown that AZT can downregulate the expression of several bacterial genes, as shown in *P. aeruginosa* [34].

The increase in the expression of cells treated with PAβN alone or AZT/PAβN combinations can be explained by the ability of bacteria to respond to stressful conditions by changing their patterns of gene expression [35]. Thus, it is possible that when the bacteria were exposed to PAβN for a prolonged period of time (6 h of treatment), efflux pumps were inhibited, causing cellular stress, which upregulated efflux pump genes in order to overcome the effect of the inhibitor. Furthermore, PAβN acts by another mechanism, which is OM disruption; thus, it could be treated by the bacteria as an insult, inducing higher expression of efflux pump genes in an attempt to rid itself of this toxic molecule. Furthermore, when the efflux pump is inhibited by PAβN, bacteria try to upregulate the regulatory genes, leading to higher expression to compensate for the inhibition of efflux pumps. In agreement with our study, another study reported a slight increase in the gene expression of *acrAB* after exposure to PAβN [36]. In the latter study, the use of efflux inhibitors did not lead to a compensatory increase in the expression of the other efflux pump genes in *E. coli*, highlighting the role of the AcrAB-TolC system as a major efflux system in *E. coli.* The same study showed that PAβN acted as a competitive substrate for AcrB and as an inhibitor of antibiotic efflux, but caused no change in the transcriptional activity of the efflux pump genes [36]. The MDR efflux complex AcrAB tightly binds to the TolC channel, potentially creating a direct link between the cytoplasm and the outer membranes [37]. In our study, *tolC* expression was significantly higher in the group treated with PAβN alone, probably due to its location in the OM, as it was directly exposed to the treatment, thus causing higher gene expression as compensation for the inhibition of the TolC protein bound to the efflux pump.

Collectively, we can conclude that when efflux pumps are inactivated or inhibited, toxic cellular metabolites accumulate, ultimately triggering the upregulation of efflux pump genes to restore homeostasis [31]. Strains with high levels of resistance to AZT which did not respond to the combination treatment exhibited lower expression levels, being unique compared to strains responding to the therapy. This was also shown in the NPN efflux pump assay for the same strains. Indeed, the genotypes and phenotypes of these strains are different, which might account for the lack of response to the combined therapy.

Overall, this study sheds light on the significance of the PAβN dual mode of action by efflux pump inhibition and outer membrane permeabilization. Indeed, this is very useful against MDR and XDR strains expressing efflux pumps, as PAβN can inhibit these pumps while disrupting the OM. As for strains lacking efflux pumps or those with low expression levels, OM’s damaging effect dominates. Thus, regardless of the nature of the strain, increased influx and/or reduced efflux of antibiotics ensures high intracellular concentrations, which is a critical factor for bacterial killing. This explains the enhancement of AZT activity when it is used with PAβN, especially in high doses.

One limitation of this study is the lack of in vivo data. Overall, there is a paucity of in vivo studies testing the effect of PAβN in combination with antibiotics. In a recent study, up to 40 μg/g of PAβN combined with neomycin were injected intramuscularly into ducks infected with *Riemerella anatipestifer*. Treatment with PAβN coupled with neomycin significantly increased the survival rate and reduced the bacterial load and pathological changes in the heart, liver, and brain of treated animals [38]. Indeed, there is a need for more studies to confirm the safety and efficacy of PAβN in vivo before employing it for therapeutic purposes.

## 4. Materials and Methods

### 4.1. Bacterial Strains

The bacterial collection investigated in this study included 53 clinical strains of *E. coli* obtained from the microbiology lab in Tawam Hospital, Al-Ain, United Arab Emirates. Bacterial identification was performed in the hospital using a VITEK 2 system (BioMérieux, Craponne, France). Three additional bacterial strains obtained from the American Type Culture Collection (Microbiologics, St. Cloud, MN, USA) were included in this study, namely, ATCC 25922 as a control (for antibiotic susceptibility and gene expression), ATCC BAA-2469 (MDR strain), and CDC AR-0346 (MDR strain which is resistant to colistin due to the presence of the *mcr-1* gene). The isolates were preserved in brain heart infusion broth (MAST, Bootle, UK) with 20% glycerol and stored at −80 °C. All the strains were checked for purity before any experiments were conducted. Some of the strains used in this study had been reported in our previous publication [16], but we used them here for different synergy experiments.

### 4.2. Antibiotic Susceptibility Testing

Antibiotic susceptibility testing was conducted following the Clinical Laboratory Standards Institute (CLSI) guidelines, using *E. coli* ATCC25922 as a quality control [39]. The disk diffusion test was used for the assessment of susceptibility to different antibiotics, including amoxicillin/clavulanate, cefpodoxime, ceftazidime, cefotaxime, cefepime, cefoxitin, aztreonam, gentamicin, piperacillin/tazobactam, ciprofloxacin, ertapenem, meropenem, and co-trimoxazole. Antibiotic disks were obtained from MAST, UK, and were applied on Mueller–Hinton agar plates (Oxoid, Basingstoke, Hampshire, UK). The broth microdilution test was used to estimate the minimum inhibitory concentrations (MICs) of selected antibiotics, including azithromycin (AZT), phenylalanine-arginine β-naphthylamide (PAβN), and others, such as ceftazidime/avibactam. Antibiotic powders were purchased from Sigma-Aldrich, USA. Mueller–Hinton broth, obtained from Oxoid, UK, was used for the assessment of MICs, following the CLSI guidelines [39]. Multidrug resistance (MDR) was defined as non-susceptibility to at least 1 antibiotic in ≥3 and <6 antimicrobial categories, while extensive drug-resistance (XDR) was defined as non-susceptibility to at least 1 antibiotic in ≥6 antimicrobial categories [40,41].

### 4.3. Detection of Macrolide Resistance Genes and Efflux Pump Genes

DNA was extracted from pure bacterial colonies by the traditional boiling lysis method [42]. PCR was used for the detection of macrolide resistance genes encoding phosphotransferases (*mphA* and *mphB*), methylases (*ermA*, *ermB* and *ermC*), and esterase (*ereA*), using the primers and cycling conditions described previously [43]. Additionally, we screened the strains for the presence of macrolide efflux pumps (*msrA*, *msrD*, *mefA* and *mefB*) and for the three components of the AcrAB-TolC multidrug efflux pump in *E. coli* [43,44]. For the amplification of each gene, 5x Hot FIREPol^®^ Ready to Load Master Mix (Solis Biodyne, Tartu, Estonia) was used with a primer concentration of 0.2 μM for each of the forward and reverse primers. All amplifications were carried out on a Veriti 96-well thermal cycler (Applied Biosystems, Carlsbad, CA, USA). PCR products were detected on an agarose gel following electrophoresis.

### 4.4. Checkerboard Assay for Synergy

The checkerboard assay was used for testing the synergy between efflux pump inhibitors and AZT, as described previously [16]. First, we conducted a pilot study using two efflux pump inhibitors (1-naphthyl-methyl-piperazine and PAβN) on four representative strains, but as 1-naphthyl-methyl-piperazine did not work well in the combinations, it was excluded from the subsequent experiments, which included only PAβN on 29 strains. A checkerboard assay was performed using an inoculum of 5 × 10^5^ CFU/mL added into 96-well microtiter plates with serial dilutions of AZT (7 dilutions starting from 2 X MIC) and PAβN (5 dilutions starting from 32 μg/mL), and incubated overnight at 37 °C. The MICs of the drugs alone and in combination were determined as the lowest drug concentrations inhibiting bacterial growth. The fractional inhibitory concentration index (FICI), used as a measure of synergy, was determined by calculating the sum of the FICs of the tested drugs, whereby the FIC for each drug represents its MIC, used in combination, divided by the MIC of each drug alone, as follows:

FICI = (MIC_1+2_/MIC_1_) + (MIC_2+1_/MIC_2_)

MIC_1_ and MIC_2_ represent the MIC of each drug alone, and MIC_1+2_ and MIC_2+1_ represent the concentrations of drugs 1 and 2 in the combination causing growth inhibition.

PAβN lacks antibacterial activity when used alone; thus, the highest concentration tested (32 µg/mL) was considered as its MIC. As for AZT, the highest concentration used (128 µg/mL) was considered as its MIC for FICI calculation for resistant strains, while its exact MIC was used for strains with determined MIC. This was carried out following the method employed by other investigators who have tested compounds without activity [45].

Based on the FICI, the combination was considered synergistic when the FICI was ≤0.5, while an antagonistic effect was considered when the FICI was >4, and no interaction was considered when the FICI was >0.5 and ≤4 [46].

### 4.5. Time-Kill Studies

Combinations showing synergistic activity according to the checkerboard assays were tested further for their killing kinetics in four selected strains, following the method described previously [16]. Briefly, bacteria were grown on tryptone soy agar (TSA) overnight at 37 °C; then, pure colonies were suspended in saline to a density of 10^8^ CFU/mL (~0.5 MacFarland) and diluted in Mueller–Hinton broth (Oxoid, UK) to a density of 5 × 10^5^ CFU/mL. Drugs were added into the appropriate wells, as described for the checkerboard assay, and incubated at 37 °C. Aliquots were collected at different time points (0, 2, 4, 6, and 24 h) and serially diluted for the enumeration of colony-forming units (CFU) by checking the growth on TSA. Two independent experiments (duplicates) were conducted for each strain, and a graph was plotted using log_10_ bacterial concentration at each time point for each concentration tested. Log_10_ CFU/mL was plotted on the Y axis against time on the X axis [47,48].

### 4.6. Outer Membrane Permeabilization Assay

The outer membrane permeabilization induced by PAβN alone and in the combinations was tested by the 1-N-phenylnaphthylamine (NPN) assay [49]. Bacterial suspensions were prepared by using mid-logarithmic phase cells at a density of 10^9^ CFU/mL (~3.0 MacFarland). These bacterial suspensions were then added to a black 96-well microplate containing 10 μM NPN (Sigma Aldrich, St. Louis, MO, USA). Serial dilutions of PAβN, colistin (positive control), AZT, or a combination of PAβN and AZM (at FICI) were subsequently added to the wells and incubated for 1 h at 37 °C, protected from light. Then, the fluorescence intensity was measured using an Infinite M200 PRO fluorescence microplate reader (Tecan, Männedorf, Switzerland) at excitation and emission wavelengths of 350 and 420 nm, respectively. NPN uptake (%) was calculated for each strain as described previously [16,50].

### 4.7. Transmission Electron Microscopy to Examine the Outer Membrane

Transmission electron microscopy (TEM) was used to examine ultrastructural changes after treatment for two selected bacterial strains (EC477 and EC500), as described in our previous publication [16]. Briefly, bacteria were suspended in LB broth (Invitrogen, Waltham, MA, USA) and incubated overnight at 37 °C. The overnight culture was diluted at a ratio of 1:30 in LB broth and kept in a shaking incubator for 2 h. The culture density was adjusted to an OD_600_ of 0.5 (~4 × 10^8^ CFU/mL), then used in the experiments, wherein bacteria were treated with either AZT (128 µg/mL), PAβN (64 µg/mL), or a combination of both at the same mentioned concentration. An untreated culture was used as a growth control (negative control), and a culture treated with colistin (2 µg/mL) was used as a positive control. Both the treated and untreated cultures were incubated for 1 h with shaking at 37 °C. The cells were harvested by centrifugation at 4 °C to avoid cell damage, washed, and resuspended in 0.1 M phosphate-buffered saline (pH 7.2). Overnight fixation of the cells was carried out using 4% formaldehyde mixed with 1.25% glutaraldehyde, 4% sucrose, 0.01 M CaCl_2_, and 0.075% ruthenium red in PBS. Following fixation, cells were washed three times with PBS, post-fixed in 1% osmium tetroxide with 0.075% ruthenium red for 1 h, and washed with water afterward. Dehydration of the cells was performed with ascending ethanol grades (30, 50, 70, 80, 90, 95, and 100%), each for 15 min, and finally treated with propylene oxide. The cells were infiltrated for 1 h each in propylene oxide/Agar 100 epoxy resin in ratios of 1:1, 1:2, and 1:3, then polymerized at 65 °C for 24 h. Blocks were trimmed, and then semithin and ultrathin sections were cut with an EM UC7 Ultracuts ultramicrotome (Leica, Vienna, Austria). Semithin sections (1.5 µm thick) were stained with 1% aqueous toluidine blue on glass slides at 70 °C. Ultrathin gold-colored sections (95 nm) were collected on 200 mesh copper grids, air-dried, and then contrasted with uranyl acetate followed by lead citrate. Finally, grids were examined with a transmission electron microscope (Tecnai Biotwin Spirit G2, Maastricht, The Netherlands) and images were captured at 80 Kv.

### 4.8. Fluorescent Assays to Detect Efflux Pump Activity and Inhibition after Treatment

1-N-phenylnaphthylamine (NPN) was also used in this assay to determine the efflux pump activity and response to treatment with PAβN. This assay is useful for the discrimination between intracellular and extracellular localization of NPN to reflect the activity of efflux pumps [51]. Previous investigators reported the use of the same method [5,52], which was followed with slight modifications. A mid-logarithmic-phase bacterial suspension was adjusted to a density of 10^9^ CFU/mL (~3.0 MacFarland) in a buffer made of 50 mM K_2_HPO_4_ and 1 mM MgSO_4_, then added into black 96-well microplates containing 10 μM NPN, along with serial dilutions of PAβN, colistin, and CCCP (both used as positive controls for outer membrane damage and efflux inhibition, respectively). The microplates were incubated at 37 °C for one hour, then properly covered to protect them from light. Efflux was initiated by adding glucose (final concentration of 1 mM), and measurements were recorded at time 0; after 10, 20, and 30 s; then every minute (from 1–5 min) using an infinite M200 PRO fluorescence microplate reader (Tecan) with excitation/emission wavelengths of 350/420 nm.

### 4.9. Gene Expression Study of AcrAB-TolC Multidrug Efflux Pump in Response to Treatment

Real-time PCR (rt-PCR) was used to test the expression of the *acrA*, *acrB*, and *tolC* genes in response to treatment with AZT and PaβN, both alone and in combination.

For RNA extraction, bacterial cells were grown at a density of 10^8^ CFU/mL (~0.5 MacFarland) in 2 mL Mueller–Hinton broth (Oxoid, UK), then treated with either AZT (2 X MIC), PAβN (fixed at 128 µg/mL), or a combination of both for 6 h. In parallel, untreated cells were incubated for the same duration for use as control. Cells were collected by centrifugation, and RNA was extracted from the pellet using a TRIzol™ Max™ Bacterial RNA Isolation Kit (Invitrogen, USA). RNA extraction started with a 5 min pre-treatment of the bacteria with Max™ Bacterial Enhancement Reagent, which contains chemicals for denaturing bacterial proteins and deactivating endogenous RNases. Subsequent bacterial lysis was performed by a TRIzol™ Reagent, which was followed by separating the aqueous solution containing the RNA by adding chloroform, precipitating the RNA from the aqueous phase using isopropanol, washing the RNA pellet with 75% ethanol to remove impurities, and finally dissolving it in nuclease-free water. The quantity and quality of RNA were evaluated using a NanoDrop™ 1000 Spectrophotometer (Thermo Scientific, Waltham, MA, USA).

Prior to reverse transcription, RNA was treated with RQ1 RNase-free DNase (Promega, Madison, WI, USA) to remove any DNA contamination from the RNA samples. Reverse transcription was performed using 1 μg of total DNase-treated RNA with FIREScript RT cDNA Synthesis Mix (Solis BioDyne, Tartu, Estonia), as recommended by the manufacturer. Reverse transcription reactions were carried out in a Veriti 96-well thermal cycler (Applied Biosystems, USA) as follows: primer annealing at 25 °C for 10 min; reverse transcription at 37 °C for 30 min; and enzyme inactivation at 85 °C for 5 min [48]. Then, cDNA was used for quantifying the expression levels of efflux pump genes (*acrA*, *acrB*, and *tolC*), using primers obtained from a previous publication [44]. Ribosomal S12 protein (*rpsL* gene) was used as a housekeeping gene (endogenous control). The reaction mixture consisted of 0.2 μM primers, 2 μL of cDNA, and 4 μL of HOT FIREPol^®^ EvaGreen^®^ qPCR Supermix (Solis BioDyne, Tartu, Estonia) with molecular biology water, for a total reaction volume of 20 μL. Real-time PCR was carried out using the 7500 Real-Time PCR System (Applied Biosystems, Carlsbad, CA, USA), with an initial denaturation step at 95 °C for 12 min, followed by 40 cycles of denaturation (95 °C—15 s), annealing (60 °C—20 s), and extension (72 °C—30 s), and, finally, melt curve analysis. Each sample was tested in duplicate.

Cycle threshold (Ct) values were used for the calculation of ΔCt using mean CT values for each gene subtracted from mean Ct values of the housekeeping gene of the same sample. Expression levels were calculated using the ΔΔCt method and normalized to the expression of the untreated control [53].

### 4.10. Statistical Analyses

IBM SPSS software (version 26, SPSS Inc., Chicago, IL, USA) was used for statistical analyses. The Kruskal–Wallis test or Mann–Whitney U-test was performed to compare the groups as was appropriate. Statistical significance was determined at *p* < 0.05. Graphs were generated using GraphPad Prism^®^ Version 9.4.1 (GraphPad Software, Inc., La Jolla, CA, USA). R Project for Statistical Computing software (R version 4.1.2) was used for dendrogram generation and heatmap construction for gene expression.

## 5. Conclusions

Efflux-mediated antibiotic extrusion is an important contributor to the resistance phenotype in many bacteria, including *E. coli.* This study elucidated the potent activity and mechanism of action of PAβN on *E. coli* by dual efflux pump inhibitory action and perturbing the OM barrier. PAβN was successful in re-sensitizing the bacteria to AZT in a dose-dependent manner. The ability of PAβN to exert its potentiating effect with AZT was also dependent on the genotypes of the strains, as PAβN/AZT combinations were ineffective in strains expressing drug target-modifying enzymes. Structural defects in the OM, as in the *mcr-1* harboring strain, can further improve OM’s destructive activity of PAβN. The success of synergetic combinations in eradicating MDR bacteria, as shown in this study, is a promising for overcoming AMR. Molecules such as PAβN, with multiple targets and modes of action, are attractive for antimicrobial drug discovery. These molecules can be used as adjuvants in conjunction with standard antibiotics, giving hope to the possibility of extending their spectrum of activity and lengthening the period of effective use.

It is recommended to test PAβN with other adjuvants in combination with other classes of antibiotics to explore its potential for enhancing the antibacterial activity of multiple antibiotics. More research efforts are required to better understand the fundamentals of drug efflux mechanisms. Owing to the multifactorial benefits of PAβN in combating MDR bacteria, in vivo studies are required to test the efficacy of PAβN with antibiotics in experimental infections in animal models before using this molecule for therapeutic purposes. Further kinetic, genomic, and transcriptomic studies are required in order to investigate the impact of efflux pump inhibitors on multiple species of MDR bacteria. Indeed, this can open the door for the discovery of more potent inhibitors of efflux pumps that can be utilized for the potentiation of antibiotics, especially on MDR bacteria, which are increasing in prevalence globally.

## Supplementary Materials

The following supporting information can be downloaded at: https://www.mdpi.com/article/10.3390/ijms24108662/s1.
