# Supplementary material for: Boosting the Antibacterial Activity of Azithromycin on Multidrug-Resistant Escherichia coli by Efflux Pump Inhibition Coupled with Outer Membrane Permeabilization Induced by Phenylalanine-Arginine β-Naphthylamide
Source: Int J Mol Sci. 2023 May 12;24(10):8662. doi: 10.3390/ijms24108662 (PMC10218020; doi:10.3390/ijms24108662)
Supplement: Supplementary file 1 [file ijms-24-08662-s001.zip › Figures S1-S3.pdf]

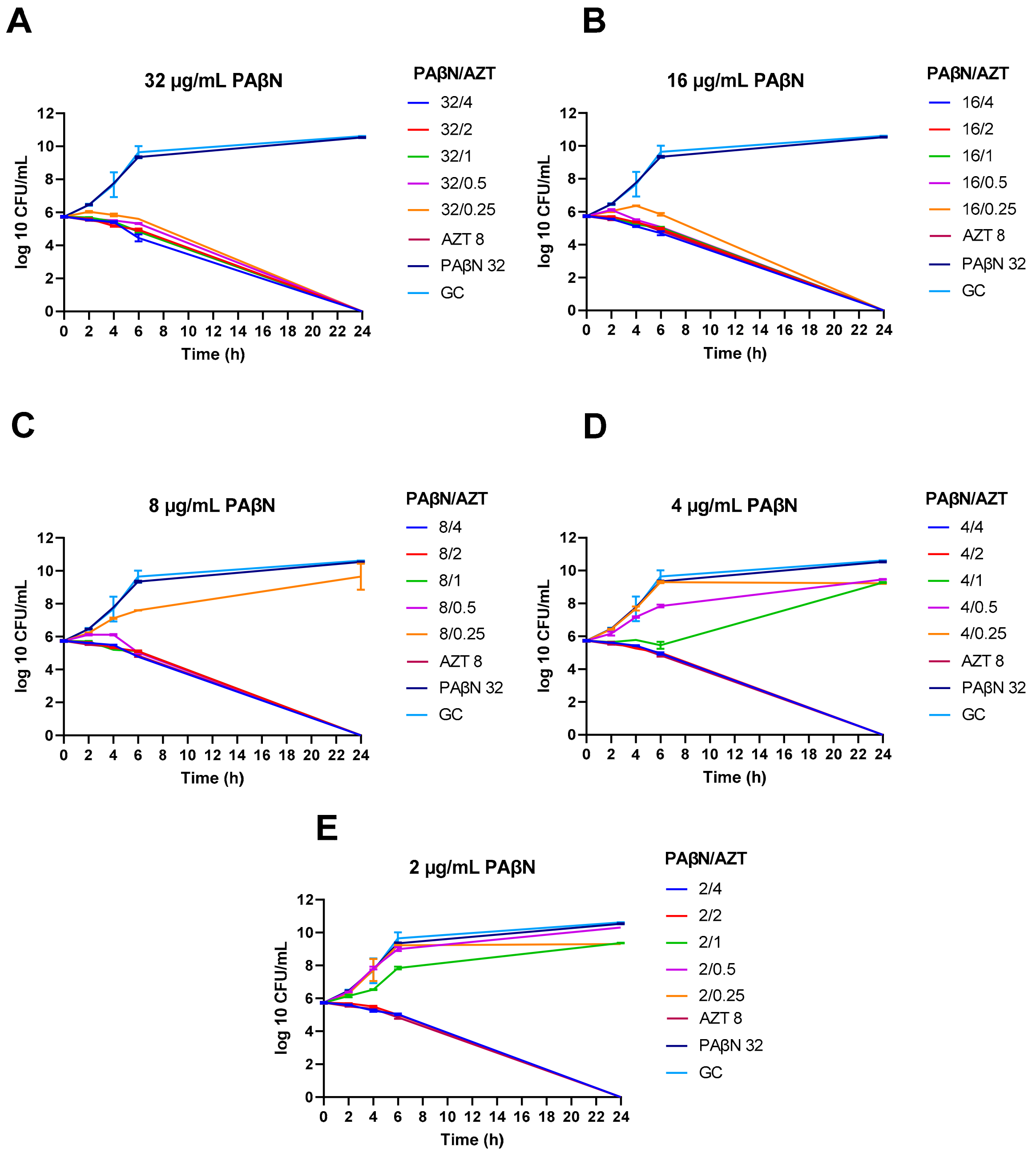

Figure S1. Synergistic effect of PAβN with AZT demonstrated by time-kill graphs for *E. coli* strain EC2. Different synergistic combinations (A-E) of PAβN (2-32 µg/mL) and AZT (0.25-4 µg/mL) were tested. PAβN (32 µg/mL) and AZT (8 µg/mL) were used as controls in addition to an untreated growth control (GC). PAβN had no antibacterial activity when used alone, while synergistic combinations with AZT ≤ ½ X MIC killed the bacteria, depending on the concentration of PAβN used. Data shown represents the mean value of duplicates from two independent experiments ± SD.

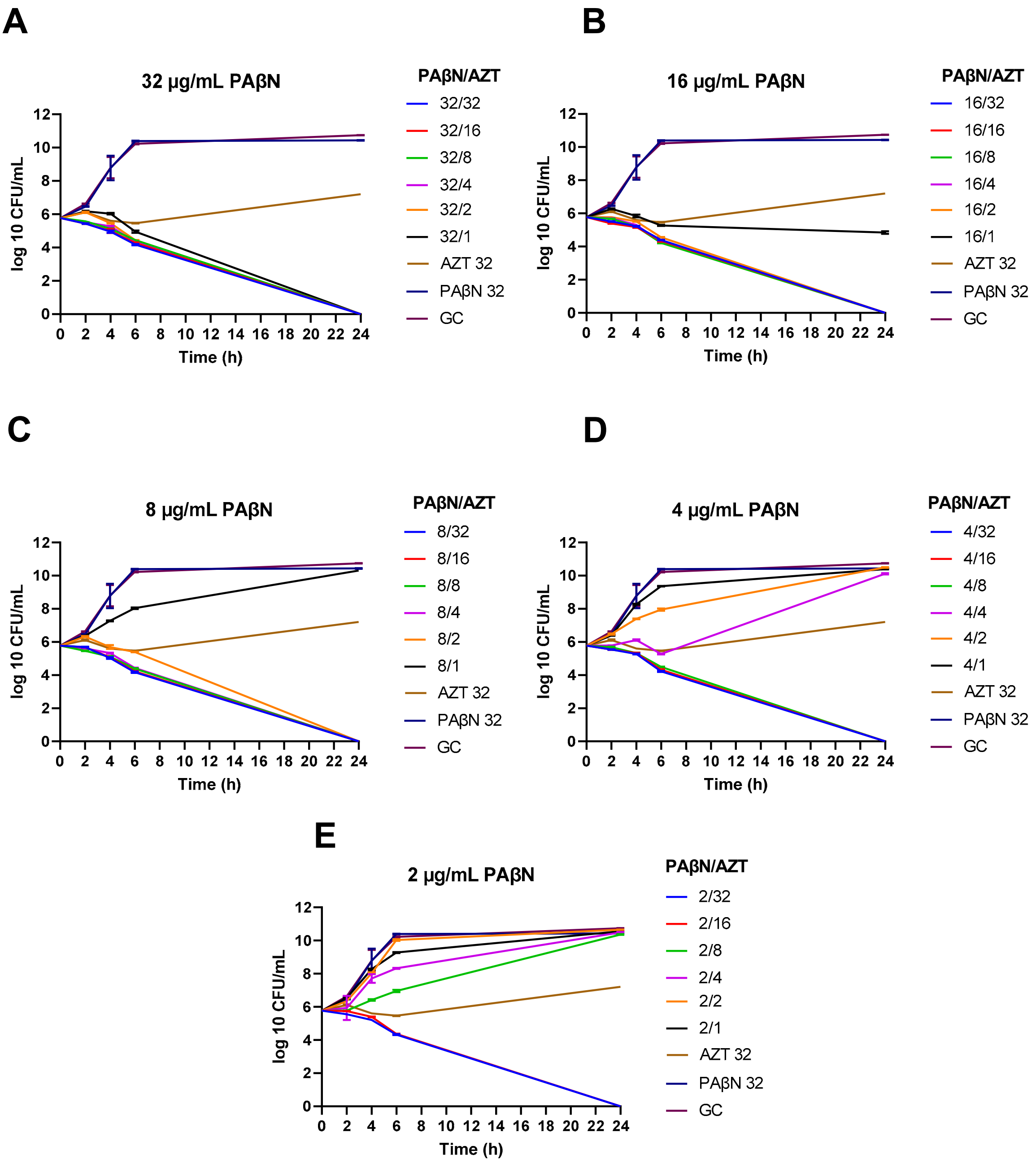

Figure S2. Synergistic effect of PAβN with AZT demonstrated by time-kill graphs for *E. coli* strain EC14. Different synergistic combinations (A-E) of PAβN (2-32 µg/mL) and AZT (1-32 µg/mL) were tested. PAβN and AZT (32 µg/mL) were used as controls in addition to an untreated growth control (GC). PAβN had no antibacterial activity when used alone, while synergistic combinations with AZT ≤ ½ X MIC killed the bacteria, depending on the concentration of PAβN used. Data shown represents the mean value of duplicates from two independent experiments ± SD.

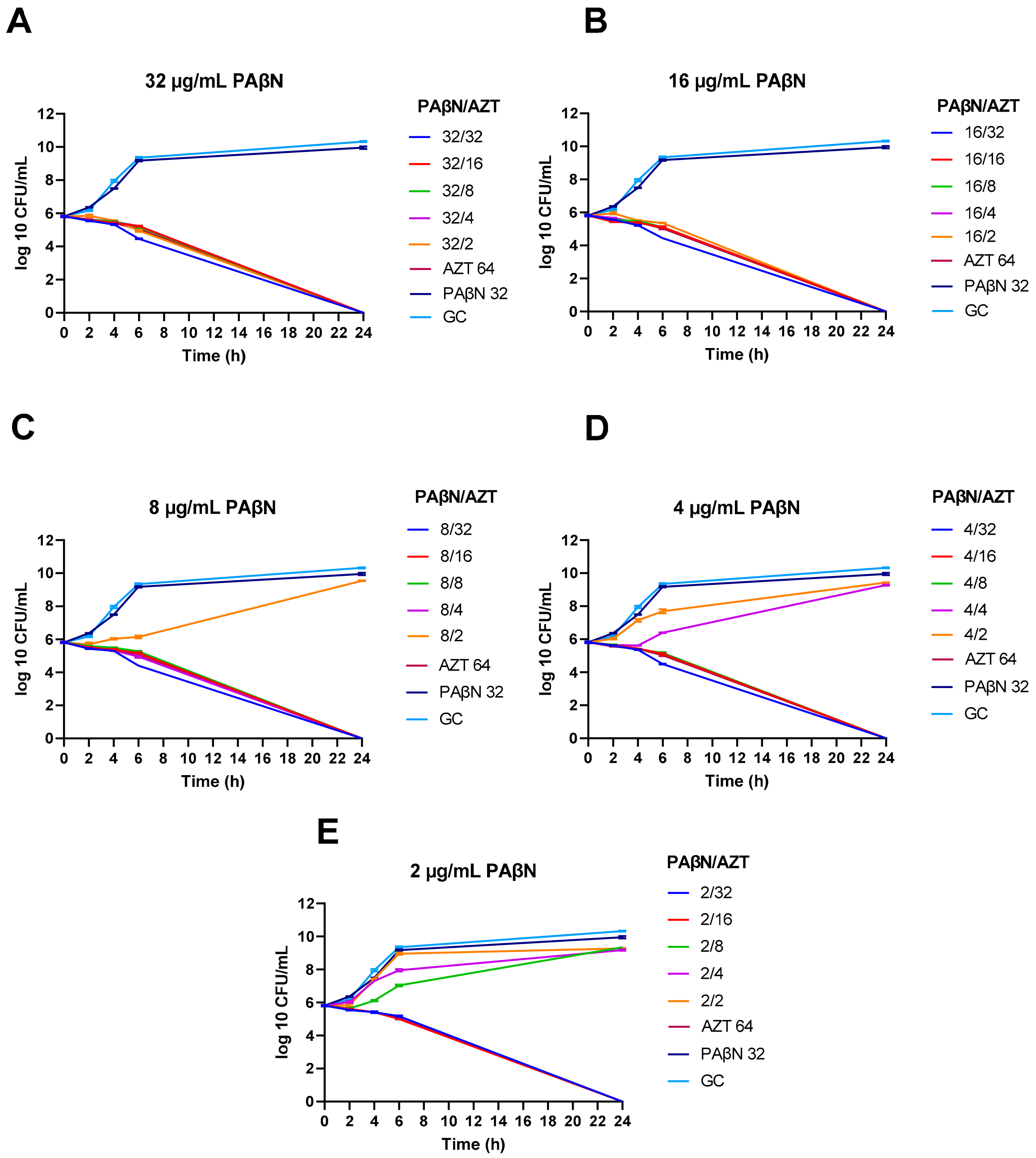

Figure S3. Synergistic effect of PAβN with AZT demonstrated by time-kill graphs for *E. coli* strain EC477. Different synergistic combinations (A-E) of PAβN (2-32 µg/mL) and AZT (2-32 µg/mL) were tested. PAβN (32 µg/mL) and AZT (64 µg/mL) were used as controls in addition to an untreated growth control (GC). PAβN had no antibacterial activity when used alone, while synergistic combinations with AZT ≤ ¼ X MIC killed the bacteria, depending on the concentration of PAβN used. Data shown represents the mean value of duplicates from two independent experiments ± SD.
